# Hydroxychloroquine Mitigates the Production of 8-Isoprostane and Improves Vascular Dysfunction: Implications for Treating Preeclampsia

**DOI:** 10.3390/ijms21072504

**Published:** 2020-04-03

**Authors:** Rahana Abd Rahman, Padma Murthi, Harmeet Singh, Seshini Gurungsinghe, Bryan Leaw, Joanne C. Mockler, Rebecca Lim, Euan M. Wallace

**Affiliations:** 1 Department of Obstetrics and Gynaecology, School of Clinical Sciences, Monash University, Monash Medical Centre, Clayton, Victoria 3168, Australia; seshini.gurusinghe@monash.edu (S.G.); joanne.mockler@monash.edu (J.C.M.); rebecca.lim@hudson.org.au (R.L.); 2 The Ritchie Centre, Hudson Institute of Medical Research, Clayton, Victoria 3168, Australia; harmeet73@gmail.com (H.S.); bryan.leaw@hudson.org.au (B.L.); 3 Department of Obstetrics and Gynaecology, Faculty of Medicine, National University of Malaysia, Kuala Lumpur 56000, Malaysia; 4 Department of Obstetrics and Gynaecology, University of Melbourne, Parkville, Victoria 3052, Australia

**Keywords:** hydroxychloroquine, preeclampsia, sFlt-1, sEng, TNF-α, endothelial dysfunction

## Abstract

In preeclampsia, widespread maternal endothelial dysfunction is often secondary to excessive generation of placental-derived anti-angiogenic factors, including soluble fms-like tyrosine kinase-1 (sFlt-1) and soluble endoglin (sEng), along with proinflammatory cytokines such as tumour necrosis factor-α (TNF-α) and activin A, understanding of which offers potential opportunities for the development of novel therapies. The antimalarial hydroxychloroquine is an anti-inflammatory drug improving endothelial homeostasis in lupus. It has not been explored as to whether it can improve placental and endothelial function in preeclampsia. In this in vitro study, term placental explants were used to assess the effects of hydroxychloroquine on placental production of sFlt-1, sEng, TNF-α, activin A, and 8-isoprostane after exposure to hypoxic injury or oxidative stress. Similarly, human umbilical vein endothelial cells (HUVECs) were used to assess the effects of hydroxychloroquine on in vitro markers of endothelial dysfunction. Hydroxychloroquine had no effect on the release of sFlt-1, sEng, TNF-α, activin A, or 8-isoprostane from placental explants exposed to hypoxic injury or oxidative stress. However, hydroxychloroquine mitigated TNF-α-induced HUVEC production of 8-isoprostane and Nicotinanamide adenine dinucleotide phosphate (NADPH) oxidase expression. Hydroxychloroquine also mitigated TNF-α and preeclamptic serum-induced HUVEC monolayer permeability and rescued the loss of zona occludens protein zona occludens 1 (ZO-1). Although hydroxychloroquine had no apparent effects on trophoblast function, it may be a useful endothelial protectant in women presenting with preeclampsia.

## 1. Introduction

Preeclampsia complicates 3%–5% of all pregnancies and remains one of the leading causes of maternal and perinatal morbidity and mortality [1]. In particular, pregnancies complicated by early onset preeclampsia (prior to 34 weeks gestation) are associated with a 20-fold increase in maternal death [2] and considerably increased rates of maternal and perinatal morbidities [3]. As such, the management of early onset preeclampsia continues to pose significant challenges to obstetricians attempting to balance maternal risks with fetal benefits of prolonging pregnancy.

Although not fully understood, the pathophysiology of preeclampsia is generally agreed to originate with poor placentation [4]. Inadequate trophoblast invasion and failure of maternal spiral arterial remodeling leads to impaired placental development, including exposure to chronic progressive ischaemia–reperfusion injury characterized by evidence of excessive oxidative stress. In turn, this induces excessive placental release of anti-angiogenic factors such as soluble fms-like tyrosine kinase-1 (sFlt-1) and soluble endoglin (sEng), coupled with inflammatory cytokines including tumour necrosis factor-α (TNF-α) and activin A [5,6,7,8]. These various factors target the maternal vasculature and contribute significantly to the widespread maternal vascular dysfunction, which is often associated with oxidative injury [9,10,11,12]. Dysfunctional cells of the vasculature are characterized by increased endothelial cell permeability, altered distribution of endothelial junctional proteins, and reduced endothelium-dependent relaxation [13,14]. 

Antimalarials, such as hydroxychloroquine, were first formally used as a treatment for cutaneous lupus in 1894. Following the observation in the 1940s that they had improved rheumatoid arthritis, they became a popular therapy in rheumatic diseases [15]. However, research has only recently unraveled some of the mechanisms of hydroxychloroquine’s therapeutic effect, as observed by Wallace et al. 2012 [16]. Hydroxychloroquine is classified as C under the U.S. Food and Drug Administration pregnancy category because it crosses the placenta but has not been reported to cause any teratogenic effects to the fetus [17,18]. It has both anti-inflammatory and immunomodulatory properties [19,20,21], and is widely used in autoimmune disorders such as systemic lupus erythematosus (SLE), rheumatoid arthritis (RA), and Sjogren’s syndrome. The exact mechanisms by which hydroxychloroquine improves the activity of these disorders are still not fully understood. However, in women with SLE, it has been shown to decrease circulating levels of pro-inflammatory cytokines IL-6, IL-8, and TNF-α [22], as well as IL-17 and IL-22, which are cytokines produced by helper T cells [23]. Recently, in a female mouse model of SLE, it was reported that hydroxychloroquine decreased endothelial oxidative stress by reducing Nicotinanamide adenine dinucleotide phosphate (NADPH) oxidase activity, which led to improved endothelial function, lower blood pressure, and a reduction in proteinuria [24]. 

In preeclampsia, NADPH oxidase-dependent oxidative stress is one of the pathways underlying the maternal endothelial dysfunction [12]. Accordingly, we hypothesize that hydroxychloroquine may confer beneficial effects in women diagnosed with preeclampsia by reducing placental production of potentially deleterious mediators, thus improving the overall maternal endothelial homeostasis. 

## 2. Results

### 2.1. Effects of Hydroxychloroquine on Placental Secretion

**Figure 1:**
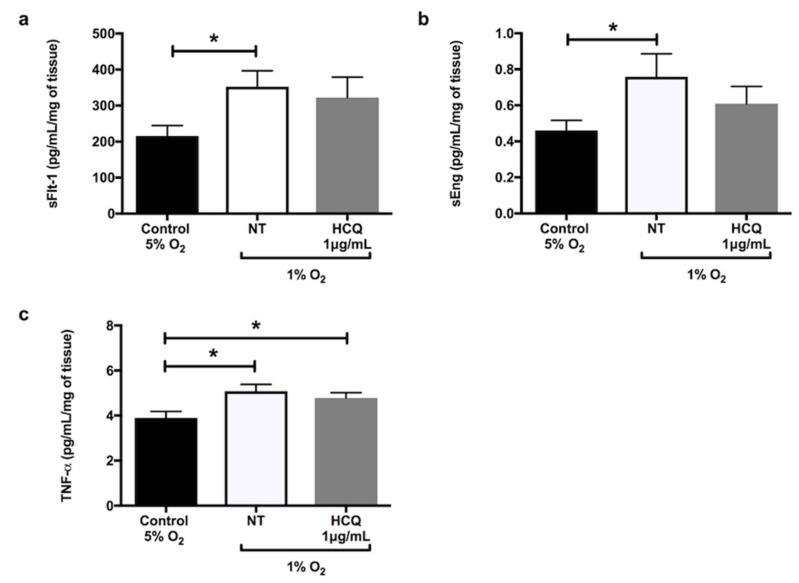
Release of (**a**) soluble fms-like tyrosine kinase-1 (sFlt-1), (**b**) soluble endoglin (sEng), and (**c**) tumour necrosis factor-α (TNF-α) by placental explants of human term normal pregnancy placentae after 24 h incubation at 5% oxygen concentration (normoxia) versus 1% oxygen (hypoxia). The explants were incubated in the hypoxic environment in the absence or presence of 1 μg/mL hydroxychloroquine. Data are mean ± standard error of the mean (SEM) from 10 independent biological replicates. * denotes *p* < 0.05. NT: non treated, HCQ: hydroxychloroquine.

**Figure 2:**
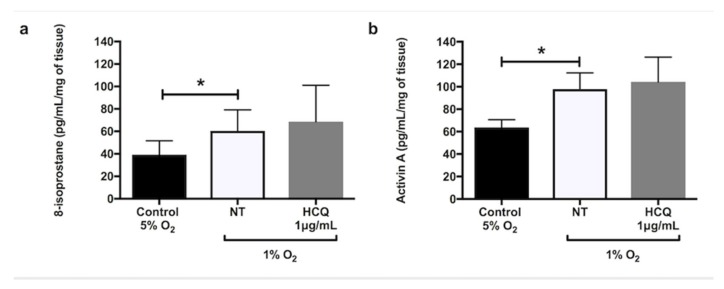
Release of (**a**) 8-isoprostane and (**b**) activin A by placental explants of human term normal pregnancy placentae after 48 h incubation at 20% oxygen concentration with 5% CO_2._ The explants were incubated in media containing xanthine (2.3 mM) + xanthine oxidase (15 mU/mL) in the absence or presence of 1 μg/mL hydroxychloroquine. Data are mean ± SEM from 10 independent biological replicates. * denotes *p* < 0.05. X/XO: xanthine/xanthine oxidase, HCQ: hydroxychloroquine.

Hypoxia significantly increased the secretion of sFlt-1 (Figure 1a, *p* = 0.02), sEng (Figure 1b, *p* = 0.02), and TNF-α (Figure 1c, *p* = 0.02) from explant cultures after 24 h incubation. In the presence of X-XO (xanthine/xanthine oxidase system), explants cultured for 48 h significantly increased secretions of 8-isoprostane (Figure 2a, *p* = 0.03) and activin A (Figure 2b, *p* = 0.01) compared to controls. Co-incubation with 1 μg/mL hydroxychloroquine did not alter either the hypoxia-induced secretion of sFlt-1 (Figure 1a), sEng (Figure 1b), or TNF-α (Figure 1c), or the X-XO-induced increase in 8-isoprostane (Figure 2a) and activin A (Figure 2b).

### 2.2. Effect of Hydroxychloroquine on HUVEC Viability

Previously we have demonstrated that, compared to untreated controls, there was no effect of hydroxychloroquine on human umbilical vein endothelial cell (HUVEC) viability across a dose range of 0.1, 1, and 10 μg/mL over 120 h in culture [25]. However, treatment of cells with 100 μg/mL hydroxychloroquine significantly reduced cell viability at 24 h (*p* < 0.001) [25]. Dosing of hydroxychloroquine for all subsequent experiments were based on these results.

### 2.3. Effects of Hydroxychloroquine on Endothelial Function In Vitro

**Figure 3:**
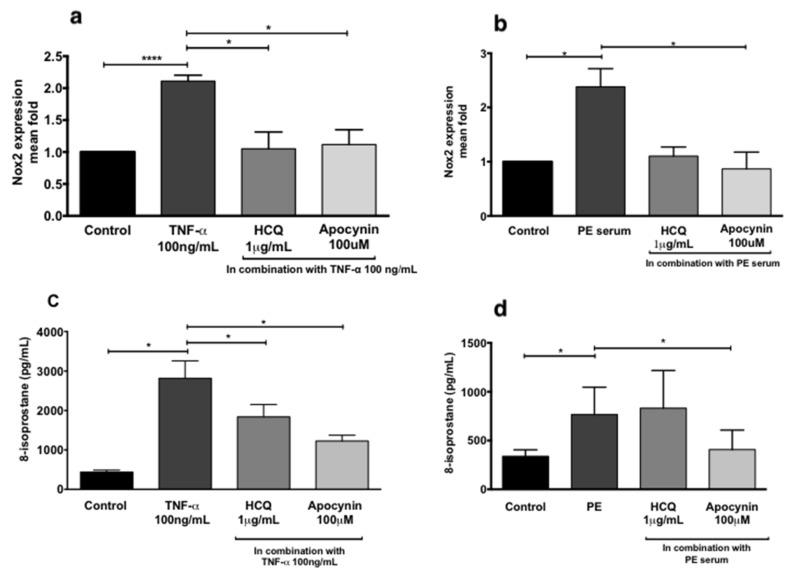
NADPH oxidase 2 (NOX2) RNA expression of human umbilical vein endothelial cells (HUVECs) treated with 100 ng/mL TNF-α (**a**) and 20% preeclampsia (PE) sera (**b**). Release of 8-isoprostane by HUVECs treated with 100 ng/mL recombinant TNF-α (**c**) and 20% preeclampsia sera (**d**). Data are mean ± SEM from eight independent biological replicates. * denotes *p* < 0.05; ****p<0.001.

HUVECs were treated in the absence or presence of (i) TNF-α (100 ng/mL), (ii) sera from normal pregnancies (20%), or (iii) sera from preeclamptic women (20%) in the presence or absence of hydroxychloroquine (1 μg/mL) to assess endothelial dysfunction (Figure 3). Compared to controls, incubation of HUVECs with TNF-α (Figure 3a,c) or sera from preeclamptic women (Figure 3b,d) significantly increased both NADPH oxidase 2 (NOX2) mRNA expression (*p* < 0.001 and *p* = 0.01, respectively) and 8-isoprostane secretion (*p* = 0.02 and *p* = 0.04, respectively). Co-treatment of HUVECs with TNF-α and hydroxychloroquine significantly reduced NOX2 mRNA expression (Figure 3a, *p* = 0.03) and secretion of 8-isoprostane (Figure 3c, *p* = 0.04). Co-treatment of HUVECs with serum from preeclamptic women and hydroxychloroquine did not significantly alter the expression of NOX2 mRNA or 8-isoprostane. However, 100 μM apocynin, a NOX inhibitor, significantly reduced the NOX2 mRNA expression and 8-isoprostane release induced by serum from preeclamptic women (Figure 3b,d, respectively, *p* < 0.01 for both).

**Figure 4:**
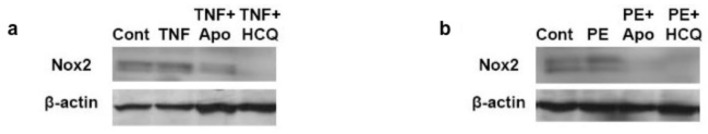
Western blot representative for NOX2 protein expression of HUVECs untreated (cont) or treated with 100 ng/mL TNF-α (**a**) or 20% preeclampsia (PE) sera (**b**) with or without apocynin (apo, 100 μM) or hydroxychloroquine (HCQ, 1 μg/mL). β-actin was used as a loading control.

Compared to controls, incubation of HUVECs with TNF-α (Figure 4a) or 20% sera from preeclamptic women (Figure 4b) increased immunoreactivity for NOX2 protein. Once again, co-treatment of HUVECs with TNF-α and either apocynin or hydroxychloroquine reduced immunoreactive NOX2 protein expression (Figure 4a). Similarly, co-treatment of HUVECs with sera from preeclamptic women and either apocynin or hydroxychloroquine also showed reduced immunoreactive NOX2 protein expression (Figure 4b).

### 2.4. Effect of Hydroxychloroquine on Vascular Permeability

**Figure 5:**
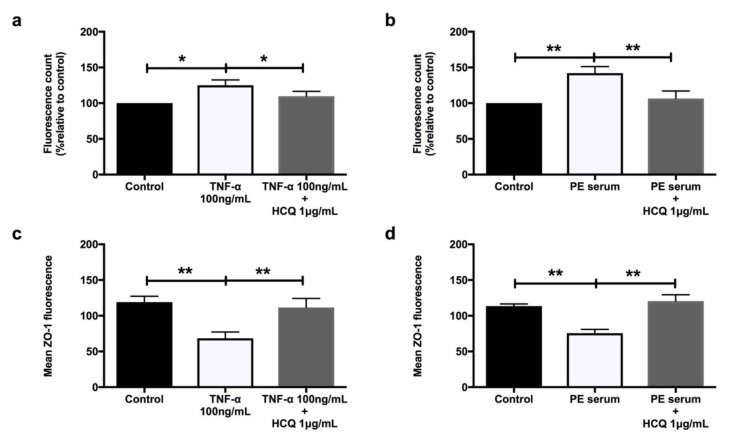
HUVECs’ permeability when treated with 100 ng/mL recombinant TNF-α (**a**) and 20% preeclampsia sera (**b**) (*n* = 9). Mean zonula occludens 1 (ZO-1) fluorescence when treated with 100 ng/mL recombinant TNF-α (**c**) and 20% preeclampsia sera (**d**) (*n* = 6). Data are means ± SEM from nine and six independent biological replicates, respectively. * denotes *p* < 0.05, and ** denotes *p* < 0.005.

Both TNF-α (Figure 5a) and sera from preeclamptic women (Figure 5b) significantly increased HUVEC monolayer permeability compared to controls (*p* = 0.02 and *p* = 0.005, respectively). These effects were mitigated by co-treatment with hydroxychloroquine (*p* = 0.04 and *p* = 0.007, respectively). Hydroxychloroquine prevented the significant loss of zonula occludens 1 (ZO-1) induced by both TNF-α (Figure 5c, *p* = 0.003) and sera from preeclamptic women (Figure 5d, *p* = 0.02).

### 2.5. Effect of Hydroxychloroquine on Zonula Occludens 1 (ZO-1) Immunohistochemistry

**Figure 6:**
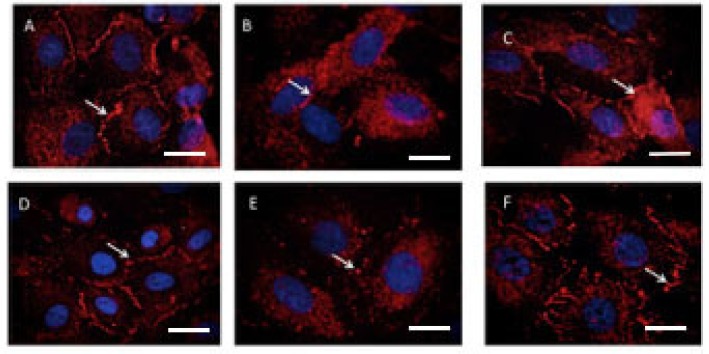
Immunofluorescent staining of ZO-1 on HUVECs treated with 100 ng/mL recombinant TNF-α or 20% preeclampsia sera for 16–22 h. Representative images from one of six experiments are shown. (**A)** Control untreated HUVECs, (**B**) TNF-α 100 ng/mL, (**C**) TNF-α 100 ng/mL with hydroxychloroquine 1 μg/mL, (**D**) control HUVECs treated with 20% normal pregnancy sera, (**E**) 20% preeclampsia sera, and (**F**) preeclampsia sera with hydroxychloroquine 1 μg/mL. Arrows show the ZO-1 staining on the endothelial cell border.

Figure 6 contains representative images of ZO-1 immunostaining. There was normal ZO-1 immunostaining in untreated or HUVECs treated with sera from normal pregnancies (Figure 6A,D), with the loss of immunostaining in cells treated with either TNF-α (Figure 6B) or preeclampsia sera (Figure 6E). Hydroxychloroquine rescued the loss of ZO-1 induced by both TNF-α (Figure 6C) and preeclampsia sera (Figure 6F).

## 3. Discussion

We undertook the study to explore the potential of hydroxychloroquine as a novel targeted therapy addressing key pathophysiological pathways in preeclampsia. We demonstrated that this antimalarial drug affords no apparent protection against hypoxia or oxidative stress in placental explants but that it does have endothelial protective properties. These observations suggest that hydroxychloroquine is a potential therapy for women with established preeclampsia but is unlikely to be useful as a preventative treatment.

We hypothesized that hydroxychloroquine would protect placental tissue from hypoxia-induced injury ex vivo. Specifically, we sought to show that hydroxychloroquine could mitigate the effects of hypoxia and hyperoxia on the placental release of the anti-angiogenic factors sFlt-1 and sEng, as well as on the release of the pro-inflammatory cytokines, TNF-α, and activin A. However, we found this not to be the case. Hydroxychloroquine had no effect on modulating hypoxia-induced placental injury. These findings support those of others who tested hydroxychloroquine in a trophoblast-derived cell line exposed to antiphospholipid antibodies as a model of antiphospholipid syndrome [26]. They found that although hydroxychloroquine was able to mitigate trophoblast secretion of IL-6, it had no effect on sEng release [26]. Collectively, this suggests that in an established diagnosis of preeclampsia, the use of hydroxychloroquine may not confer any beneficial effects. 

The maternal symptoms of preeclampsia are largely due to widespread maternal endothelial dysfunction [27,28]. Lupus shares this feature as the key mechanism underlying hypertension, renal dysfunction, and other organ injury [29]. Indeed, the endothelial dysfunction in both preeclampsia and lupus have also been shown to be due, at least in part, to excessive oxidative stress secondary to NOX activation [12,30,31]. Recently, in murine models of lupus, hydroxychloroquine was shown to reverse endothelial dysfunction via the downregulation of NOX, and subsequently, oxidative stress [24,32]. Here, we showed that hydroxychloroquine may have similar effects in an in vitro model of preeclampsia-like endothelial dysfunction. Specifically, hydroxychloroquine was able to prevent the TNF-α induction of NOX2 and subsequent oxidative stress in HUVECs but, importantly, was not able to block similar effects induced by sera from preeclamptic women. Interestingly, apocynin, a NOX inhibitor, was able to prevent the effects of both TNF-α and sera from preeclamptic women on NOX2. This confirms the pro-oxidative effects of the sera of preeclamptic women are mediated via NOX2 [12]. Müller-Calleja et al. demonstrated the inhibition of reactive oxygen species (ROS) generated by endosomal NOX in human monocytic cells [33]. However, the concentration used was much higher (10 µM) but still within therapeutic range as compared to ours (3.6 µM). It is apparent that the concentration of hydroxychloroquine differs in various cell types in in vitro experiments. Although several isoforms of NOX family members including NOX1, NOX2 (also called gp91phox), NOX3, NOX 4, and NOX 5 have been reported to date, in endothelial cells, NOX1, NOX2, and NOX4 isoforms are reported to be involved in the inflammatory response and cytokine expression, triggered by angiotensin II treatment, through different mitogen activated protein kinases (MAPK) pathway activation (phosphorylated form of p38 MAPK, extracellular signal regulated kinases, ERK-1/2 and stress-activated protein kinases and c-jun N-terminal kinase, SAPK/JNK) [34].

We have shown before that follistatin, an activin binding protein, can block the endothelial effects of sera from preeclamptic women [12,35]. Compared to women with a normal pregnancy, maternal circulating levels of activin are increased approximately 10-fold in women with preeclampsia [36]. We have not yet explored whether hydroxychloroquine can block activin-mediated effects. However, the current study suggests that sera from preeclamptic women contains factors capable of inducing NOX, which cannot be mitigated by hydroxychloroquine.

Intriguingly, hydroxychloroquine was able to mitigate the effects of both TNF-α and sera from preeclamptic women on the loss of endothelial ZO-1 and integrity. Endothelial ZO-1 is a protein present in endothelial cell–cell junction known to regulate cellular permeability [37]. Any changes in ZO-1 protein, such as induced by a response to inflammatory cytokines, will alter the endothelial cell permeability. The loss of ZO-1 induced by TNF-α is known to be mediated via the activation of NOX [38,39]. Hydroxychloroquine was able to prevent the loss of ZO-1 and the subsequently increased endothelial permeability induced by both TNF-α and sera from preeclamptic women, suggesting these effects may be mediated through a TNF-α-dependent upregulation of NOX. Further evaluation is required to verify this theory, perhaps using TNF-α receptor antagonists co-incubated with the sera of preeclamptic women.

Circulating levels of TNF-α increase in normal pregnant women and are further raised in preeclamptic women [40,41,42]. These levels are much lower than the concentrations tested in the present study (15 pg/mL vs. 100 ng/mL, respectively). Although in the in vitro model of acute exposure of cells to single high dose may not be a good representation of the in vivo situation, previous studies have reported that 100 ng/mL of TNF-α in cultured HUVEC enhanced endothelial cell activation, similar to that observed in preeclampsia [43].

The effect of hydroxychloroquine on other pathogenic pathways of preeclampsia have not been explored in this study. For example, it is now thought that a key mechanism of action of antimalarial drugs is the antagonism of toll-like receptor (TLR) signaling and subsequent downstream activation of pro-inflammatory cytokines [16,44]. In preeclampsia, placental expression of TLR3, TLR7, and TLR8 are upregulated [45,46]. The treatment of pregnant rodents with TLR agonists induces a preeclampsia-like phenotype, providing further evidence of other mechanistic pathways that hydroxychloroquine treatment could also target. 

In addition to the effects of antimalarial agents on TLRs, these drugs have other benefits, such as inhibition of phospholipase A2 (PLA2) enzyme. PLA2 has been implicated in the pathogenesis of preeclampsia and is found to be elevated in both decidual tissue and the sera of preeclamptic women [47,48]. Similarly, in patients with active SLE, there is a 4.6-fold increase in the mean activity of PLA2 [49]. Lipid peroxidation occurs because of oxidative stress induced by the elevated levels of reactive oxygen species. This leads to membrane phospholipid degradation and hence release of arachidonic acid [50]. Zabul et al. have described the potential role of arachidonic acid hydroperoxide underlying the molecular mechanism of oxidative stress in preeclampsia [51]. Arachidonic acid stimulates release of superoxide from neutrophils and macrophages [52]. Antimalarial drugs have been shown to inhibit PLA2 activity and therefore reduce the generation of superoxide, which will be beneficial for improving endothelial dysfunction in preeclamptic patients [50,53].

## 4. Materials and Methods 

### 4.1. Blood and Tissue Collection

Preeclampsia is defined as elevation of blood pressure of 140/90 mmHg or more, and proteinuria of more than 0.3 g in a 24 h urine collection or random urine dipstick test of more than 2+ according to the Society of Obstetric Medicine of Australia and New Zealand guidelines [54]. All blood and placental tissues were collected from pregnant women, as detailed below. Written and informed consent was obtained from all individual participants included in the study with the approval of the Monash Health Human Research Ethics Committee (HREC no. 13357B, dated 6 August 2015). Venous blood was collected from women with a singleton healthy pregnancy and from women with established preeclampsia at 24 to 34 weeks of gestation. Women who had received intravenous magnesium sulphate, or had pre-existing or secondary hypertension, diabetes, or a multiple pregnancy, were excluded. None of the women with preeclampsia were in labor at the time of blood sampling. The control (healthy) women were matched for gestation (≤34 weeks). Sera were separated and pooled into two groups: healthy term pregnancy serum and preeclampsia serum. For all in vitro experiments, 20% pooled sera from preeclampsia pregnancies were used for treatment of endothelial cells and were compared with that of the normotensive sera treated cells. There were significant differences in the systolic and diastolic blood pressure and proteinuria between normotensive and preeclamptic patients (*p* = 0.007), as previously presented [25].

### 4.2. Placental Explant Cultures Ex Vivo

Placental villous explants (*n* = 10) were collected from term uncomplicated pregnancies at elective caesarean section within 20 min of delivery of the placenta. Briefly, placental villous tissue was excised by removing maternal decidua. Villous explants (approximately 50–70 mg wet weight) were then thoroughly washed with cold Hank’s balanced salt solution (HBSS, 1:10, Life Technologies/Thermo Fisher Scientific, Waltham, MA, USA) and placed in 24-well plates in M199 supplemented with 1% antibiotics/antimycotics (penicillin G, streptomycin sulphate, and amphotericin B) and 1% of L-glutamine (all from Life Technologies/Thermo Fisher Scientific, Waltham, MA, USA). Incubation details are provided below.

### 4.3. Placental Hypoxia

Placental hypoxia was modeled by incubating placental explants in 1% oxygen with 5% CO_2_ at 37°C in the presence or absence of 1μg/mL hydroxychloroquine (Sigma-Aldrich, St. Louis, Missouri, USA). Controls were incubated in 5% oxygen. The conditioned media were collected after 24 h and stored at -80°C for sFlt-1, sEng, TNF-α, and activin A assay.

### 4.4. Placental Oxidative Stress

The explants were treated with 2.3 mM xanthine (X) and 0.015 U/mL xanthine oxidase (XO) (Sigma-Aldrich) to induce oxidative stress [8,55]. Explants were incubated in X/XO in the presence or absence of 1 μg/mL hydroxychloroquine for 48 h at 37 °C in 20% oxygen, 5% CO_2._ Untreated cultures served as controls. Conditioned media were collected and stored at -80 °C in the presence of 0.005% butylated hydroxytoluene (BHT) (Sigma-Aldrich, St. Louis, Missouri, USA) to prevent autoxidation for activin A and 8-isoprostane assay measurements. Elevated levels of 8-isoprostane are a marker for lipid peroxidation caused by oxidative stress [56] and, in addition, high levels of activin A have been implicated in the pathway of placental oxidative stress [12].

### 4.5. Measurement of sFlt-1, sEng, TNF-α, and Activin A with ELISA

Levels of sFlt-1, sEng, TNF-α, and activin A were measured in placental explant (*n* = 10) conditioned media using Quantikine immunoassay ELISAs (R&D systems, Minneapolis, Minnesota, USA) according to the manufacturer’s protocol. All samples were assayed in duplicate. Briefly, for the measurement of sFlt-1, sEng, TNF-α, and activin A, the conditioned media was diluted (1:40, 1:10, 1:5, and 1:30, respectively) with assay diluent. Results were normalized per milligram weight of tissue.

### 4.6. Human Umbilical vein Endothelial Cell (HUVEC) Isolation 

Umbilical cords were also obtained from healthy women with term singleton pregnancies (*n* = 8) undergoing elective caesarean. HUVECs were isolated and cultured, as previously described, with minor modifications [8,57]. Briefly, the umbilical cord was severed from the placenta within an hour of collection. All areas with clamp marks were removed and the umbilical vein was cannulated and tied with thread. After removal of blood, the umbilical veins were infused with type II collagenase (0.5 mg/mL, Sigma-Aldrich) and incubated for 10 min at 37°C to isolate the endothelial cells. They were maintained in M199 complete media containing 20% heat-inactivated fetal calf serum, 1% antibiotics/antimycotics (penicillin G, streptomycin sulphate, and amphotericin B), and 1% l-glutamine with endothelial and fibroblast growth factor (10 ng/mL each). Only cells at passage 2 to 4 were used for experiments.

### 4.7. HUVEC Viability Assay 

We first determined the effect of different concentrations of hydroxychloroquine on HUVEC viability. Cells were plated at 2 × 10^4^ cells per well in 96-well plates (*n* = 8, Corning) and grown to confluence in 100 μL culture media with hydroxychloroquine added at different concentrations (0.1, 1, 10, 100 μg/mL) and further incubated for 24 h. Viability was assessed by adding 20 μL MTS (3-(4,5-dimethylthiazol-2-yl)-2,5-diphenyltetrazolium bromide) reagent (Promega, Madison, WI, USA) to each well. After 1 h at 37 °C, the absorbance at 490 nm was read using a plate reader (SpectraMax i3, Molecular Devices, San Jose, CA 95134 USA). 

### 4.8. Oxidative Stress as Assessed by 8-Isoprostane

Cells were grown to confluence in 96-well plates (2 × 10^4^ cells per well) for 24 h in M199 complete media. Cells were treated with media (control), 100 ng/mL TNF-α (Life Technologies /Thermo Fisher Scientific, Waltham, MA, USA), 20% normal pregnancy sera, or 20% preeclampsia sera, in the presence or absence of hydroxychloroquine (0, 0.1, 1, and 10 μg/mL) for a further 24 h. Conditioned media were then stored at -80°C in the presence of 0.005% butylated hydroxytoluene (BHT) as described above. Total 8-isoprostane was measured using a commercial enzyme immunoassay (Cayman Chemical, Ann Arbor, MI, USA) according to the manufacturer’s instructions. Samples were assayed in duplicate after diluting 1:5 with assay diluent. On the basis of the results from this experiment, in all subsequent experiments 1 μg/mL hydroxychloroquine was used. The cells were treated with either 100 ng/mL of recombinant TNF-α or 20% preeclampsia sera in combination with either 1 μg/mL hydroxychloroquine or 100 μM apocynin (NADPH oxidase inhibitor) (Sigma-Aldrich) for 24 h.

### 4.9. Measurement of NADPH Oxidase (NOX2) mRNA Expression

Cells were grown to confluence in 6-well plates (1 × 10^5^ cells per well) for 48–72 h in M199 complete media. Cells were treated with 100 ng/mL recombinant TNF-α or 20% preeclampsia serum combined with either 100 μM apocynin or 1 μg/mL hydroxychloroquine for 6 and 12 h, respectively. The treatment groups were compared with untreated HUVECs or cells treated with 20% sera from normotensive pregnant women. Total cellular RNA was isolated with Ambion (Life Technologies /Thermo Fisher Scientific, Waltham, MA, USA) according to the manufacturer’s protocols. The cDNA was prepared with 1 μg of cellular mRNA and reverse-transcribed using SuperScript III first strand synthesis system (Life Technologies). Quantitative PCR was performed on Rotorgene (Qiagen, Hilden, Germany) in a reaction mixture (20 μL) containing Sensimix SYBR Green PCR master mix (Bioline Meridian Biosciences, Heidelberg, Germany). The reactions were performed with the following conditions: 95 °C for 10 min then 40 cycles of 95°C for 20 s, 60 °C for 30 s, and 72 °C for 30 s. NOX2 was amplified using primers 5′-TGG CAC CCT TTT ACA CTG-3′ and 5′-CCA CTA ACA TCA CCA CCT CA-3′. The housekeeping gene 18S was amplified using primers 5′-GTC TGT GAT GCC CTT AGA TGT C-3′ and 5′-AAG CTT ATG ACC CGC ACT TAC-3′. Relative gene expression was determined using the delta delta – cycle threshold (CT) method. 

### 4.10. Measurement of NOX2 Protein Expression

HUVECs were grown to confluence and treated with either 100 ng/mL recombinant TNF-α or 20% preeclampsia serum combined with 100 μM apocynin or 1 μg/mL hydroxychloroquine for 6 and 12 h, respectively. HUVECs were assessed for total NOX2 protein. Protein extracts of nucleic and cytoplasmic fractions were obtained using the nuclear and cytoplasmic reagents (Life Technologies /Thermo Fisher Scientific, Waltham, MA, USA) according to manufacturer’s instructions. Protein quantification was performed using the Pierce Bicinchoninic acid (BCA) kit (Life Technologies /Thermo Fisher Scientific, Waltham, MA, USA)). For Western blots, 40μg protein was loaded for each sample. Membranes were then blocked with 5% (*w/v*) skim milk in phosphate-buffered saline with 0.1% (*v/v*) Tween-20 for 1 h prior to probing with antibodies. Membranes were stripped in a mild stripping buffer (1.5% *w/v* glycine, 0.1% *w/v* sodium dodecyl sulfate, 1% *v/v* Tween-20 in distilled water) for 5 min between antibodies. The primary antibodies and concentrations used were Nox2 at 0.025 ng/mL (anti-NOX2/gp91phox antibody (ab80508, Abcam, Cambridge, United Kingdom) and the control β-actin at 0.01 ng/mL (IMG-5142A, Imgenex). Antibodies were diluted in blocking buffer and incubated overnight at 4 °C. Chemilumiscence detection was performed using Clarity Western Electrochemiluminescence (ECL) blotting Substrates (Bio-Rad, Hercules, CA, USA).

### 4.11. Endothelial Permeability Assay

An endothelial permeability assay was performed as previously described with minor modifications [58]. Briefly, culture inserts (0.4 μm pore size, 6.5 mm diameter; Corning) were coated with 0.2% gelatin (Sigma-Aldrich) for 30 min at room temperature. HUVECs (50,000 cells per well) were plated on the inserts and cultured to form a tight monolayer with 100 μL M199 complete media in the upper chamber and 600 μL in the lower chamber at 37 °C, 5% CO_2_ for 72 h. Inserts were then transferred to a fresh plate and cell monolayers were treated in fresh media containing 100 ng/mL recombinant TNF-α alone or with 1 μg/mL hydroxychloroquine for 16–22 h. Treatment groups were compared with untreated HUVECs. The conditioned media were collected and 100 μL fresh media containing fluorescein isothiocyanate (FITC)-conjugated dextran (MW 40000, final concentration 1 mg/mL, Sigma-Aldrich) was added to the upper chamber. The plate was incubated while protected from light for 60 min. The media from the lower chamber were diluted (1:20) in HBSS for measurement of fluorescence at 485/535 nm using a plate reader (SpectraMax i3, Molecular Devices). Results (fluorescence units) were expressed as percent changes relative to control.

Assessment of cell permeability when treated with 20% sera from healthy or preeclamptic women was performed using in vitro permeability assay kit from Millipore (Merck Millipore) in the absence or presence of 1 μg/mL hydroxychloroquine for 16–22 h. The treatment groups were compared with HUVECs treated with serum from women who had normal pregnancies (NP). Briefly, the transwells, which were coated with collagen, were rehydrated with 250 μL endothelial growth media (EGM, Lonza) and left at room temperature for 15 min. Subsequently, 200 μL of the media was removed and replaced with an equal volume of cell stock (1 x 10^5^). Then, 500 μL of media was added to the receiver plate and incubated for 72 h to form a tight monolayer. Following this, fresh media was replaced in the receiver plate. The cells were treated accordingly and further incubated for 16–22 h. Media in the upper chamber was replaced with fresh media (150 μL) containing fluorescein isothiocyanate (FITC)-conjugated dextran, and the plate was incubated for 30 min and protected from light. The media from the lower chamber was diluted (1:20) with HBSS for measurement of fluorescence at 485/535 nm using a plate reader (SpectraMax i3, Molecular Devices). Results (fluorescence units) were expressed as percent changes relative to control.

### 4.12. Zonula Occludens (ZO-1) Immunohistochemistry for the Assessment of Endothelial Integrity

HUVECs were grown on 14 mm glass coverslips (4 × 10^4^ cells/well) placed in 24 well plates. Cells were treated with 100 ng/mL recombinant TNF-α or 20% sera from preeclamptic women in the presence or absence of 1 μg/mL hydroxychloroquine for 16–22 h prior to fixing with 4% paraformaldehyde (Sigma-Aldrich) for 30 min at room temperature. The treatment groups were compared with untreated HUVECs or cells treated with 20% normal pregnancy sera. Cells were blocked with 0.5% bovine serum albumin (BSA, Sigma-Aldrich) for 30 min, incubated first with rabbit anti-ZO-1 (1:50, Zymed) overnight at 4°C, then with donkey anti-rabbit Alexa Fluor 568 (1:100, Invitrogen) for 1 h in the dark. Cell nuclei were stained with 2 μm 4′,6-diamidino-2-phenyindole dilactate (DAPI, Sigma Aldrich) for 10 min and mounted with fluorescent mounting media (DakoCytomation). Staining was examined with an Olympus BX60 fluorescent microscope and images were taken using an Olympus DP70 camera and Olympus CellSens software (Olympus). The primary antibody was replaced with an isotype-matched control antibody in the negative controls. The mean intensity of the staining was assessed using ImageJ software (version 2.0.0-rc-43/1.50i, http://imagej.net/Fiji/Downloads, Bethesda, MD).

## 5. Conclusions

Hydroxychloroquine has a significant protective impact on endothelial function acting via the suppression of NOX-induced oxidative stress; it is unable to mitigate all of the effects of preeclamptic sera-induced injury in vitro or to mitigate ex vivo placental injury. Further evaluation is warranted to determine other molecular pathways by which hydroxychloroquine may prevent endothelial dysfunction in preeclampsia. The results of this study strongly suggest that hydroxychloroquine seems likely to be clinically effective as adjuvant therapy in women diagnosed with preeclampsia.
